# Movement predictability of individual barn owls facilitates estimation of home range size and survival

**DOI:** 10.1186/s40462-022-00366-x

**Published:** 2023-02-07

**Authors:** Shlomo Cain, Tovale Solomon, Yossi Leshem, Sivan Toledo, Eitam Arnon, Alexandre Roulin, Orr Spiegel

**Affiliations:** 1 School of Zoology, Faculty of Life Sciences, Tel Aviv University, 69978 Tel Aviv, Israel; 2 Blavatnik School of Computer Science, Tel Aviv University, 69978 Tel Aviv, Israel; 3 Department of Ecology and Evolution, Building Biophore, University of Lausanne, 1015 Lausanne, Switzerland

**Keywords:** Animal personality, Behavioral plasticity, Behavioral syndromes, Bio-telemetry, Double-hierarchical generalized linear model (DHGLM), High-throughput tracking, Movement ecology

## Abstract

**Background:**

There is growing attention to individuality in movement, its causes and consequences. Similarly to other well-established personality traits (e.g., boldness or sociability), conspecifics also differ repeatedly in their spatial behaviors, forming behavioral types (“spatial-BTs”). These spatial-BTs are typically described as the difference in the mean-level among individuals, and the intra-individual variation (IIV, i.e., predictability) is only rarely considered. Furthermore, the factors determining predictability or its ecological consequences for broader space-use patterns are largely unknown, in part because predictability was mostly tested in captivity (e.g., with repeated boldness assays). Here we test if (i) individuals differ in their movement and specifically in their predictability. We then investigate (ii) the consequences of this variation for home-range size and survival estimates, and (iii) the factors that affect individual predictability.

**Methods:**

We tracked 92 barn owls (*Tyto alba*) with an ATLAS system and monitored their survival. From these high-resolution (every few seconds) and extensive trajectories (115.2 ± 112.1 nights; X̅ ± SD) we calculated movement and space-use indices (e.g., max-displacement and home-range size, respectively). We then used double-hierarchical and generalized linear mix-models to assess spatial-BTs, individual predictability in nightly max-displacement, and its consistency across time. Finally, we explored if predictability levels were associated with home-range size and survival, as well as the seasonal, geographical, and demographic factors affecting it (e.g., age, sex, and owls’ density).

**Results:**

Our dataset (with 74 individuals after filtering) revealed clear patterns of individualism in owls’ movement. Individuals differed consistently both in their mean movement (e.g., max-displacement) and their IIV around it (i.e., predictability). More predictable individuals had smaller home-ranges and lower survival rates, on top and beyond the expected effects of their spatial-BT (max-displacement), sex, age and ecological environments. Juveniles were less predictable than adults, but the sexes did not differ in their predictability.

**Conclusion:**

These results demonstrate that individual predictability may act as an overlooked axis of spatial-BT with potential implications for relevant ecological processes at the population level and individual fitness. Considering how individuals differ in their IIV of movement beyond the mean-effect can facilitate understanding the intraspecific diversity, predicting their responses to changing ecological conditions and their population management.

**Supplementary Information:**

The online version contains supplementary material available at 10.1186/s40462-022-00366-x.

## Introduction

Animal movement reflects the interaction between an individual's needs and its changing environment and directly impacts its fitness and central ecological processes, such as foraging and dispersal [1, 2]. Movement patterns show remarkable diversity across different scales, varying among species, populations, individuals, and also within individuals across time [3–5]. Differences in movement among conspecifics are often related to environmental heterogeneity (e.g., in resource distribution), demographic factors (e.g., sex and age), and short-term behavioral states (e.g., hunger level; [6–9]). In addition to these well-established factors, there is a growing attention to individuality in movement and its influence on ecological patterns and population response to changing conditions. Yet, despite a surplus of recent studies, the patterns of individuality and their consequences remain relatively understudied [10–14].

Studies in behavioral ecology have long-established the framework and tools to quantify consistency in individual behaviors, and the existence of animal personalities (aka temperament or behavioral types; hereafter BTs) in traits such as boldness, exploration and sociability [15, 16]. More recently, accumulating evidence demonstrates that individuals are also repeatable in their movement and spatial behaviors, forming “spatial-BTs” [10, 17]. Examples include repeatable home-range behaviors [18, 19], habitat use [20, 21], or finer-scale movement indices like maximal daily displacement [22, 23]. The growing interest in spatial-BTs is driven in part by the ever-improving ability to track more individuals at better resolution and accuracy, providing enough data on sufficient sample size to dissect the contribution of among-individual variation from other factors like background environments [24]. These rich datasets, in turn, can reveal the impact of fine-scale movements on social interactions, disease transmission and other behaviors that may influence fitness and population dynamics and ultimately the ability of species to adapt to environmental changes [25–27].

Despite the above-mentioned immense progress in describing spatial-BTs, this is mostly done as the difference in the mean level among individuals (e.g., [17, 19, 23]). Differences among individuals in their intra-individual variation (IIV) have been thoroughly discussed for other behaviors (e.g., boldness; [28]) but are rarely considered in movement studies. IIV is often termed individual unpredictability [28–30]. It is defined as the residuals in a measured behavior after accounting for the among-individual differences in their mean response (i.e., intercepts), and for the plasticity of their response to environmental gradients (i.e. slopes, which may also differ among individuals; [28, 30–32]). For instance, Stamps et al. [28] repeatedly assayed the boldness (latency to emerge after disturbance) of hermit crabs (*Pagurus bernhardus*). They found that individual crabs differed in their mean level and had a plastic response of diminishing trend (habituation). Yet individuals also differed in their predictability around the individual-specific expected level: some were predictably closer to the expected trend while others showed high IIV around the trend-line.

Broadly speaking, predictability usually increases (IIV decreases) with age or experience (sometimes referred to as canalization; [33]). Predictability may also vary between sexes, depending on the species’ natural history. Both predictability and plasticity can be regarded as axes of BT if individuals are stable over time. Quantifying predictability (IIV) requires repeated assessments of the behavior in mind. Hence, most studies on this topic were done in captivity (e.g., [33] or [34]). Despite the methodological advantages of captive-assays, this approach is limiting the ability to link observed differences in predictability with their *in-situ* ecological consequences for individual fitness, space use, and ultimately population dynamics [35]. Exploring predictability in movement behavior can overcome these cons and offers a suitable alternative approach.

Biologging datasets may provide the required large samples of repeated measures per individual (e.g., daily routines; [24, 36]) with a balanced design and large sample sizes across individuals. Studies by Cleasby et al. [37] and Hertel and Niemela [38] also provide a methodological route to test predictability in movement by double-hierarchical generalized linear models (DHGLMs) that simultaneously capture individual variation in intercept (behavioral type), slope (behavioral plasticity), and residual variance (predictability). Recent work by Hertel and colleagues [38, 39] suggests that highly predictable individuals coexist along with unpredictable individuals within the same population, and that predictability may co-vary with movement rate creating a movement syndrome (sensu; [23]) that may have potential ecological impacts.

Ideally, quantifying predictability in movement indices requires large datasets with multiple individuals simultaneously tracked at high resolution over long periods (thus experiencing relatively similar external conditions) [32]. Indeed, empirical in situ examples are still particularly rare, at least in part due to these data requirements. Thus, it is still unknown whether spatial-BTs differ also in their predictability (or just in the mean trait), and if the predictability is potentially another relevant spatial-BT axis. Further, to the best of our knowledge, none of the previous examples tested if predictability in movement is associated with other ecological properties, such as broad-scale space-use patterns or fitness proxies (as was done for predictability of other behaviors; [35]). Similarly, it remains unknown if age and other plausible predictors (like sex, and environmental conditions) influence predictability in movement, as was shown for other behaviors ([33] and reference therein). This sets the ground for broadening the axes of individual variation by exploring also consistent differences among individuals in their movement predictability.

To address these gaps, we focused on a population of barn owls (*Tyto alba*). This nocturnal predator plays a key role as a pest control agent in agricultural areas [40]. Accordingly, barn owls’ natural history has been relatively well described [41], and their high reliance on nest boxes (which allow farmers to boost owls' local population in areas where natural nests are limited; [40]) along with ongoing monitoring facilitates captures and tracking. We have used high-resolution tracking data to quantify owls’ predictability and its ecological consequences. Specifically, we ask three broad questions (followed by corresponding hypotheses). First, what are the patterns of among-individual variation in movement? We hypothesize that [H1.1] individuals differ consistently in their movement patterns (i.e., showing a spatial-BTs in their mean levels); that [H1.2] they also differ in their predictability (IIV around the mean); and that [H1.3] these varying levels of predictability are repeatable across time (i.e., an individual that was relatively predictable over period A will be relatively predictable over period B; e.g., [35]). Second, we ask what are the ecological consequences of predictability? Since BTs are known to affect broader space use patterns and fitness [10, 42], we hypothesize that predictability may be associated with both [H2.1] home-range size and [H2.2] individual survival. Lastly, we ask what factors affect the predictability of individuals? We hypothesize that beyond possible effects of environmental factors (e.g. local density), [H3.1] age and sex will co-vary with this index, as was shown in several previous examples (limited mostly to captive behavioral assays in controlled environments; [33, 38, 43]).

## Methods

### Study species

The barn owl (*Tyto alba*) is a medium-sized bird (length 33–39 cm; wingspan 80–95 cm) from the Tytonidae family, with a cosmopolitan distribution [41]. It is known as a generalist top predator, which feeds almost exclusively on small mammals located in agricultural fields and open grasslands. At the same time, as secondary cavity breeders, barn owls are limited by the availability of suitable sites in these habitats. As a result, they tend to breed in man-made structures (e.g., barns, farmhouses, and ruins)—as their name indicates.

Barn owls have a remarkable breeding capacity. They lay large clutches ranging from 2 to 11 (e.g., ~ 4.6 fledglings in Israel; [44]). The laying interval is about two to three days, with asynchronous hatching (i.e., the female starts incubation as soon as the first egg has been laid) resulting in substantial age differences within the brood. Eggs require, on average, 32 days (range 27–36 days) of incubation. Hence, the overall incubation period varies from 34 for up to 59 days (32 for incubation of first egg + 27 days of within clutch delays) for clutches of 11 chicks. During incubation, the female usually remains inside the nest cavity, while the male provides her food requirements. When all eggs are hatched (typically about two weeks after the first one is hatched), the female starts leaving the nest more regularly. Fledglings leave the nest for the first time at approximately the age of 55 days [45], but the parental care extends for ~ 30 more days [46]. In Israel, eggs are laid around March (extending from January to June; [44]), and pairs can sometimes raise up to two broods annually [47].

### Study system

**Fig. 1:**
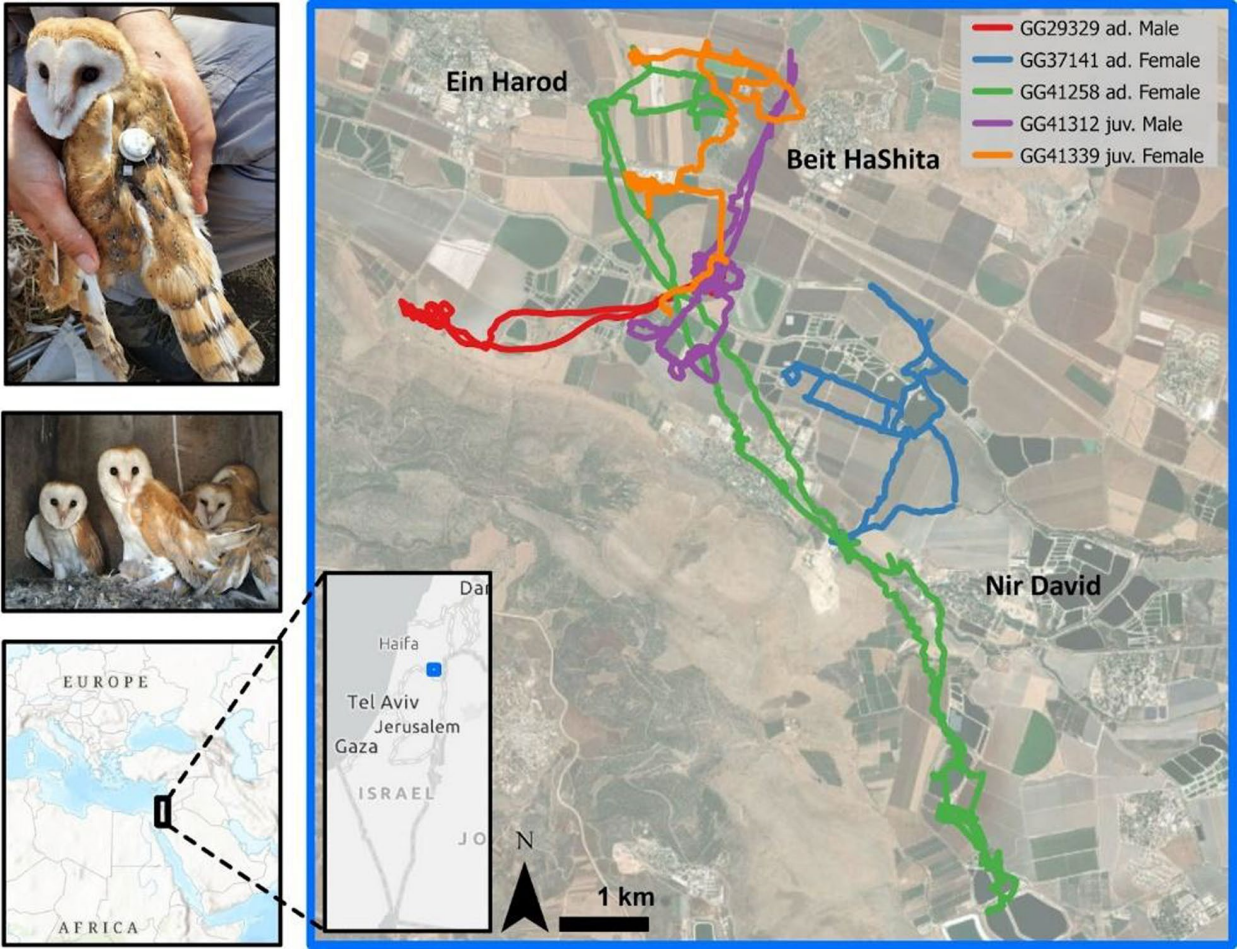
The upper-left panel shows an owl equipped with an ATLAS tag in backpack configuration. The middle left panel shows four fledglings in a nest box. The main panel shows the nightly trajectories of five independent owls (not the same ones as above) over one night in January 2022 and the insets show the regional and national location of the study site at the Harod Valley, Israel

The study was conducted at the Harod valley (32° 30' N 35° 29' E), north-eastern Israel. This valley is dominated by intensive agricultural landscapes (field crops, orchards, banana plantations and fishponds), and holds a few rural settlements. Over the last three decades, local farmers deployed dozens of nesting boxes for barn owls` usage in agricultural fields throughout the area. This effort enhances owls’ resident population and their biological service of rodents control [40]. This ongoing agro-ecological project includes monitoring nest-boxes (every breeding season) and diurnal owl captures (while roosting) by blocking the nest-box door. Captured owls are banded with metal rings for individual identification, aged and measured for standard measurements (wing length and body mass). In addition to this ongoing monitoring, from December 2019 to February 2022, we fitted tracking devices (hereafter tags) to adults and fledglings (wing > 280 mm) in good body condition (a total of 94 owls). Tags were attached with a Teflon harness in a backpack or leg-loop configuration (total weight of the device and harness was 13 ± 1 g; < 4.3% of the body mass in all cases; Fig. 1). Sex was determined for these individuals with genetic kits on feathers samples (Karnieli-Vet Ltd., Kiryat Tiv’on, Israel).

All trapping and tagging procedures were authorized by permits 2019/42155 and 2020/42502 from Israel Nature and Parks Authority.

### The ATLAS tracking system

We have used an ATLAS wildlife tracking system to address our hypotheses. In addition to the animal-borne tags, the ATLAS system includes a set of ground stations with tower-mounted antennas and central data-processing and storage servers [48–50]. The system uses a reverse-GPS approach (i.e., localizations are estimated from data received by the stations whose coordinates are known and not through onboard calculations by the tags themselves). Tags transmit radio signals with unique tag-IDs at 1/4 or 1/8 Hz (depending on settings) and these are received by ground stations. If three or more ground stations successfully receive a given signal, then the tags’ location is computed at high accuracy (ideally about ± 5 m; [50, 51]), from the times of arrival of the signals. The use of ground stations limits the coverage to a particular region (whereas GPS offers global coverage), but this is acceptable for studies of barn owls that largely remain local. Such an approach allows tags to be inexpensive, lightweight, and battery efficient, compared to other alternatives (e.g., GPS where the tag localizes itself), providing many more data points per gram. These features make the system particularly effective in collecting high resolution and accurate data for many individuals over extended periods (months-years) and hence appropriate for investigating individual variation in movement patterns and predictability.

### Data analysis and statistical methods

#### Filtering and processing

All data analyses were done in the R environment [52] with Rstudio [53]. To ensure data quality we applied several data filtering and segmentation steps. First, following a previously described pipeline [54], we filtered out points with low system-accuracy estimate (STD > 50 m) or with non-realistic speed-line > 15 m s^−1^. Second, to reflect the owl's nocturnal activity, the data was segmented into nights, starting at 5 p.m. and ending at 6:00 a.m. Daytime data was excluded, and nights were used as the base unit for all further analyses. Third, we computed the nightly movement indices (see below) only if tracking provided sufficient data points (more than 1000 points per night). Fourth, our data (female static behavior, chick ages using wing length of the oldest fledgling in the nest; [55]) allows us to back-calculate the start and end dates of the incubation period for each female and hatching dates for the fledglings. We excluded incubation periods from female movement analysis and considered the transition from fledgling to adult at the age of one year. Fifth, because barn owl behavior and movement patterns change drastically along the year, we divided the data into three periods, representing different stages in the owls’ reproductive cycle and seasonality (1st-period: Feb–May, incubating/nesting period; 2nd-period: Jun–Sep, rearing/post-breeding period; and 3rd-period: Oct–Jan, fall-winter time). We then calculated movement indices for each period excluding owls with less than 25 nights in a given period. The latter two steps reduce potential biases caused by individuals leaving our site, or age-dependent dispersal.

Several movement indices are conceivable for quantifying individual consistency, such as the nightly total-distance (i.e., the sum of all movements/flight segments in the night), trajectory openness and others [see 36, this volume]. To minimize the influence of location errors during stops, we segmented the trajectories (i.e., the daily tracks) by their activity mode, move (i.e., fly) or stop segments, and estimated total distance for moving segments only. These indices were computed using the AdpFixedPoint function from R-package toolsForAtlas. For simplicity, we focus here on results from the nightly max-displacement as the main index of movement, defined as the distance between the first point of the night (typically the nest box) to the most distant point in the nightly trajectory. This distance is both very commonly used, and robust to minute differences in the sampling interval.

#### Estimating individual repeatability

To test our initial hypothesis regarding consistent individual variation in movement [H1.1], we calculated repeatability (Rp) using Nakagawa and Schielzeth [56], as the proportion of the total variance accounted for by differences among individuals. In addition to repeatability, we also report the coefficient of variation for among-individual variance (CVi), calculated as the among-individual variance standardized by the trait mean. Both repeatability and CVi are population-level estimates of the degree of individual variation, with CVi suggested as a more robust estimate [38].

**Fig. 2:**
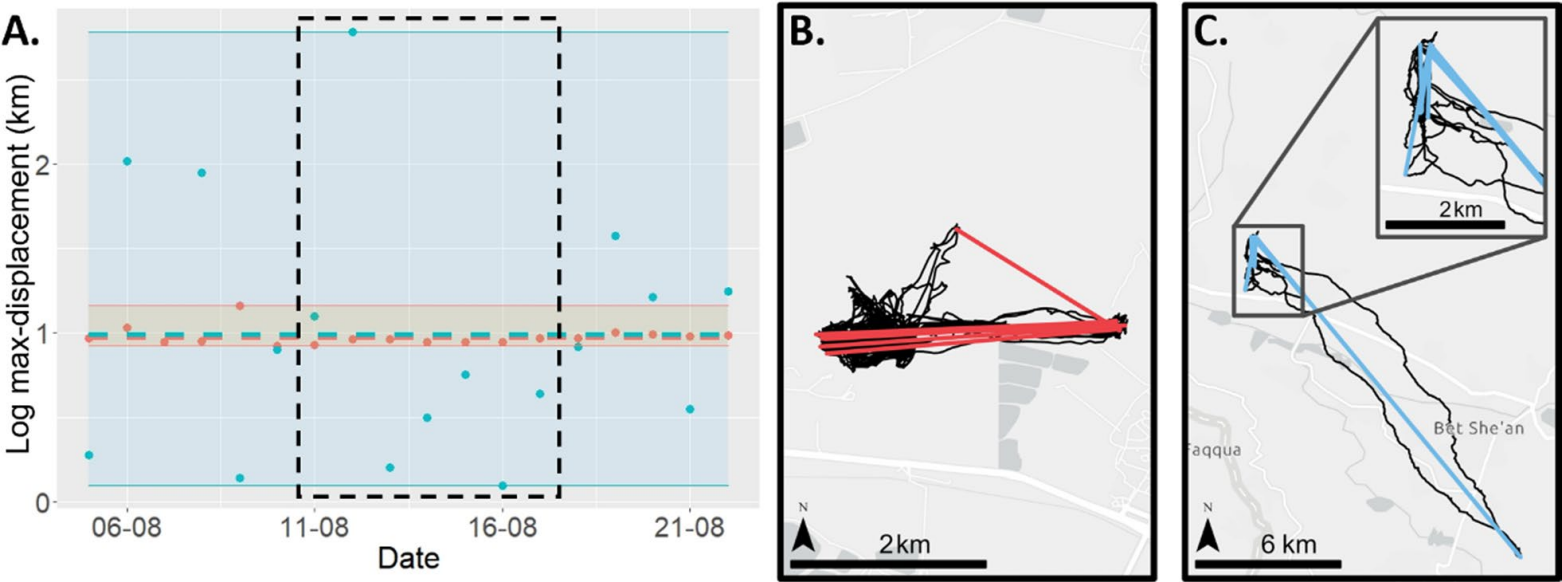
A demonstration of different predictability values. **A** Nightly maximal displacement of two individuals over three weeks during August 2021. While the two individuals have similar average max-displacement, they differ strongly in their predictability values. One individual (red) has highly consistent distances (~ 2.6 km every day), and the other (blue) varies substantially in its nightly movement range, alternating between nights of short distances and nights of up to ~ 15 km. **B** and **C** Maps showing the trajectories (black line) and relevant nightly max-displacement distances (colored red and blue lines, respectively) for eight nights within this period (marked in black dash on panel **A**) of predictable individual (**B**) and unpredictable individual (**C**)

Individuals may differ in their average nightly max-displacement (flying near or far) and also in their variability around their mean. Some individuals are unpredictable and are producing a broad range of nightly movement ranges, whereas more predictable individuals are narrowly centered around their own average (Fig. 2). We measured individual predictability [H1.2] by estimating variation in residual intra-individual variation (rIIV), i.e., the spread of residuals around an individual reaction norm (after accounting for differences in both intercept and slope) following the protocol of Cleasby et al. [37] and Hertel et al. [38]. We used the R-package brms [57] to fit a double-hierarchical generalized linear model (DHGLM) to our datasets with nightly max-displacement as a response variable. Individuals with a high residual variance in the DHGLMs are accordingly more unpredictable than individuals with a low residual variance. When necessary for biological interpretation of rIIV values we back-transformed them to the original scale (km) by taking the exponent of its logged values from the DHGLM outputs.

Finally, to determine whether individual predictability is consistent across time (H1.3; e.g., [35]), we calculated period-specific predictability values and established individual consistency also in this aspect, similarly to the calculation of repeatability and CVi of max-displacement. Then, we established the ecological relevance of individual predictability for broad topics in movement ecology by exploring the predictive power of this index on home-range (HR) estimates [H2.1] and survival [H2.2], on top and beyond commonly investigated indices of age, sex, mean max-displacement and geographical factors (detailed below) that might affect the movement.

#### Home-range analysis

We estimated the owls’ HR (the area used by an individual for its routine activities; [58]) for each period. We quantified the utility distribution function of each individual’s space-use using the auto-correlated kernel density estimate (akde; [58]) from the R-package ctmm [59]. This method is explicitly suitable for estimating these parameters from high-resolution movement data, as available here. Due to the function's long running time over our huge dataset (with tens of millions of points), for this analysis we have subsampled the data at a 10 min interval. Further, in order to represent the HR as locations where the owl actually chooses to stand (e.g., perch or rest) and not places where it flies through, we have used only the segments of activity mode identified as stops (and not flight), as described above. An individual of unknown sex and an individual whose HR model did not converge were excluded from this analysis.

We extracted the locations of HR centers (available from the ctmm output) and used these to explore the influence of geographical position on the HR size. Specifically, we included four factors: the center coordinates (longitude, latitude), ground elevation and its distance from the system’s center (to eliminate possible bias in our tracking dataset). Lastly, we also tested the effect of owls’ population density on the dependent variables using an index of the number of occupied boxes within a radius of 1.7 km (the median HR’s radius) along the study years. This index provides a robust density estimate that reflects the massive agro-ecological project and is not biased by our sampling efforts of the focal tracking.

To explore the effect of predictability on HR size [H2.1] while accounting for other factors, we compared a set of linear mixed effect models (lmer function from R-package lme4; [60]). All models included Log(HR) as the dependent variable (for a given period) and individual’s ID as a random effect. We ran a preliminary analysis that included the following fixed effects (varied among models): Three categorical factors: age (adult vs. juvenile), sex, and period; and four continuous fixed effects (standardized before inclusion): the tracking duration (number of nights) within the period, mean max-displacement, mean total-distance and mean rIIV (index of predictability of max-displacement). We checked for collinearity among fixed effects and considered models with most possible combinations, excluding a few models with singularity issues. Models were then ranked with the corrected Akaike information criterion (AICc) using R-package AICcmodavg [61]. After establishing the top three models (Delta_AICc ≤ 4.3) we also explored whether the geographical factors (Coordinates, elevation, distance from the ATLAS center) and/or the local owl density improve the models of HR size. A models ranking was conducted again. Prediction plots from the top model were then generated with the R-package effects [62].

#### Survival analysis

For testing our hypothesis that predictability affects survival [H2.1], beyond the effects of age, sex, and max-displacement we modeled survival using Cox hazard regression [63, 64]. This method is commonly used for investigating the effect of several variables upon the time a specified event (in our case, death) takes to happen. The coefficients in a Cox regression relate to hazard; a positive coefficient indicates a worse prognosis and a negative coefficient indicates a protective effect of the associated variable. An individual’s fate at the end of the tracking is logged as a “censored” status (live or unknown) or “dead”. We implemented this regression with coxph (R-package survival; [65, 66]), and used the function ggforest (R-package survminer; [67]) to present results for the model as a ‘Forest’ plot.

Various individuals died during the study (N = 22 out of 74 owls), many of them through collision with cars, a well-documented mortality factor of barn owls around the world [41, 68, 69]. We included mean max displacement, mean rIIV, sex, age group (classified as juvenile or adult for each individual) and total tracking duration (days until last known status) as predictors of survival in the model. Five individuals that transitioned between age groups during the tracking period were considered separately for each group (i.e., surviving for one year as a juvenile, and then according to their adult fate). Since our regression model does not account for the repeated measures of these five individuals, we repeated the analysis using mixed effects Cox hazard regression model (coxme; [70]). This latter version (with ring-ID used as a random factor) resulted in very similar results to that of the simple regression described above (see Additional file 1: Tables S1 and S2), and for simplicity, we present here only the simpler model.

#### Factors affecting predictability

For testing our third hypothesis regarding the factors affecting individual predictability, we have modeled rIIV (unpredictability) as a function of age group, sex, year, and period. We also accounted for the effect of local density and geographic variables (latitude, longitude, elevation, distance from center) by considering these in the models, along with the tracking duration. We constructed generalized linear mixed-model with an individual’s ID as a random factor and all possible combinations of these fixed effects using the function dredge (R-package MuMIn; [71]). We ranked the models according to their AICc values and investigated the effect size of the relevant models. Finally, to further validate this modeling approach we also modeled the predictors affecting rIIV (the residuals of the model) directly into a DHGLM. These models showed the same qualitative results as the simpler models described above (see Appendix).

## Results

We obtained data for 92 different individuals during 2019–2021, with a tracking duration of 115.2 ± 112.1 (X̅ ± SD; range: 1–570) nights per individual, and a total of over 10,600 nightly trajectories (containing > 58 × 10^6^ ATLAS fixes). After data filtering and excluding birds with insufficient data per period, our dataset includes 128 periods-bird combinations with 74 different individuals and an average of 1.7 ± 1.0 (range: 1–5) periods per individual. These birds include 45 females and 29 males of which 34 are adults and 45 are juveniles (five individuals transitioned from juvenile to adult during the tracking period). See Additional file 1: Tables S3 and S4 for full details.

Barn owls typically start their nocturnal activity at their central roosting site (often a next box) and use several perching points along the night before returning to their day roost. They pass a nightly distance (i.e., a ‘total distance’) of 11.82 ± 7.73 km (range: 2.26–51.89), with maximal displacement (i.e., a bee-line distance from the starting point to the most distant point in the night) of 2.45 ± 0.93 km (range: 0.56–5.41 km). Season has a strong effect on barn owls’ movement. During the breeding season, owls act as central place foragers, frequently returning to the nest to provide for the incubating female (the males) and later for the chicks (both parents, males often more intensively than females). After fledging and a short flight-learning period juveniles had longer distances compared to adults, and males show longer distances compared to females (one-way ANOVA, F_*df* = 3_ = 8.97, *p* < 0.001; see Additional file 1: Fig. S1).

### Individual variation in barn owl movement

**Fig. 3:**
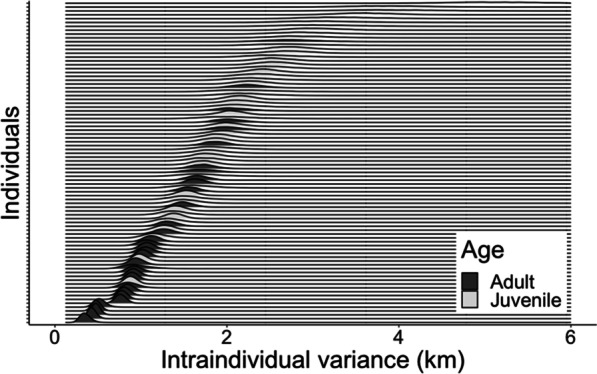
Posterior estimates for individual predictability in distances of nightly max-displacement across the entire tracking duration. Higher values of rIIV (X-axis) indicate lower predictability in the behavior. Each line on the y-axis corresponds to a different individual, sorted by their mean rIIV. The most predictable individual has an average residual variance of 0.35 km around its behavioral mean, whereas the least predictable individual has an average residual variance of 5.16 km, resulting in a very flat distribution with a high mean value. Dark color indicates adults while light color indicates juvenile birds and the apparent trend that adults are much more predictable than juveniles is supported by subsequent models, see below

In agreement with our hypothesis [H1.1], barn owls show a significant individualism in their max-displacement with repeatability of Rp = 0.228 [0.158, 0.297; confidence interval] and coefficient of variation for among-individual variance (CVi) = 0.362 [0.303, 0.426]. Individuals also differ in their overall predictability estimates (Fig. 3). Period-specific predictability values (for birds tracked in more than one period) reveal that also this trait is strongly repeatable for each individual (i.e., an individual that was predictable in period A was similarly predictable on period B), with repeatability estimated as Rp = 0.424 [0.185, 0.638]. These results were not limited to the index of max-displacement, we found quantitatively similar patterns also for the index of nightly ‘total distances’, with repeatable values and varying predictability among individuals (Additional file 1: Table S5). Overall, these findings support our second [H1.2] and third [H1.3] hypotheses regarding rIIV values being consistent individual traits, and below we turn to explore the ecological consequences of this variation and the factors that affect it.

### The ecological consequences of predictability in movement

#### Individual predictability is related to its HR size

Ranking models for HR size shows that all top models (top eleven models with a cumulative weight of 0.98), which reliably explain the data, include our index of predictability (rIIV), together with age, period, mean max-displacement, and tracking duration (Additional file 1: Table S6). The influence of the geographic factors and density on rIIV is limited, and these predictors were not included in the top model, only appearing in some of the following models (e.g., 2nd to 5th models, cumulative weight of 0.46; Additional file 1: Table S6). Accordingly, model-averaged effects for the HR size shows that both Max displacement and rIIV had the strongest effect, while the geographical variables had negligible effects. Similarly, HR sizes differed among periods, with larger sizes during rearing/post-breeding season and fall-winter compared to the incubating/nesting HRs, when individuals are bounded to their respective nests (Table 1).

**Table 1:** Model-averaged coefficients for fixed effects included in models predicting HR size of barn owls

	Estimate	Unconditional SE	95% Unconditional confidence interval
**MaxDisp**	**0.54**	**0.09**	**0.37, 0.71**
**rIIV**	**0.34**	**0.09**	**0.17, 0.51**
**Period-peri2**	**0.56**	**0.2**	**0.16, 0.96**
**Period-peri3**	**0.47**	**0.22**	**0.03, 0.9**
Age- juvenile	0.27	0.18	− 0.08, 0.61
Sex-male	0.03	0.16	− 0.26, 0.36
Tracking duration	0	0	− 0.01, 0
Density	− 0.01	0.01	− 0.02, 0.01
Elevation	− 0.07	0.08	− 0.23, 0.08
Latitude	− 0.01	0.08	− 0.16, 0.14
Longitude	− 0.09	0.08	− 0.24, 0.07
Distance from center	− 0.09	0.08	− 0.24, 0.06

Reference categories for categorical variables were Adult (age group), Female (sex) and Period 1 (tracking periods). Coefficients with significant effects are in **bold**

MaxDisp—mean nightly maximal displacement; rIIV—the index of (un)predictability in max-displacement, mean value; Tracking duration—the number of tracking nights for each period; Density—the number of occupied boxes within median HR size from the HR center whose location refers to the Longitude, Latitude coordinates, Elevation (m above sea level) and Distance from the system's center

**Fig. 4:**
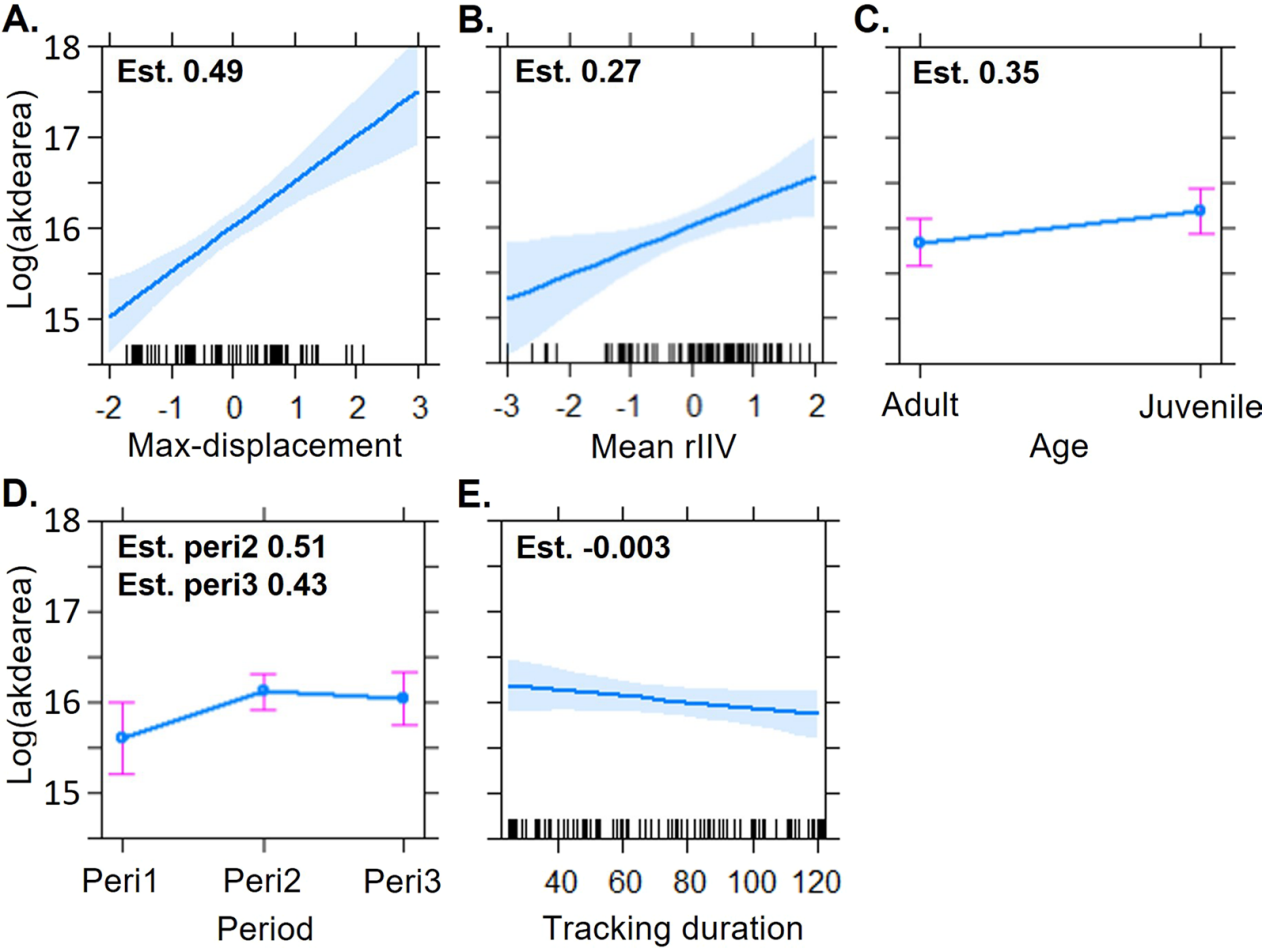
The factors affecting barn owls’ home-range (HR) according to the top-ranked model and auto-correlated kernel density estimate for HR size. Each panel presents the prediction plot for one of the factors (note that the y-axis is set on the same range for all panels): **A** Mean max-displacement (standardized); **B** rIIV of mean max-displacement (standardized); **C** Age (categorical); **D** Periods; **E** Tracking duration. Sex was absent from the predictors of the best models (see text for details)

Focusing on predicted effect sizes (Additional file 1: Table S7, Fig. 4) from the leading model demonstrates that—in agreement with our hypothesis [H2.1]—the predictability per se has a major effect on HR size, independent of all other considered factors. Expectedly, max-displacement (Fig. 4A) has the strongest effect on HR, with individuals moving further at the nightly scale having also large HR at the longer scale of a full period. More surprising is the strong effect of predictability (Fig. 4B), which equals to about a half of the former effect of the max-displacement. The positive effect indicates that individuals with higher rIIV (less predictable) have larger HRs. Also age has an expected effect with juvenile barn owls having larger HR than adults (Fig. 4C). Periods differed as well (Fig. 4D), with HR size in the 1st period (Feb-May; breeding season) being lower compared to 2nd (Jun-Sep, rearing/post-breeding period) and 3rd (Oct-Jan, fall-winter time) periods (with no clear difference among the latter two). Sex (lower ranked models) and tracking duration (which we included in all models; Additional file 1: Table S6; Fig. 4E) had very weak effects on HR size. The latter result reflects our conservative minimal inclusion criteria (at least 25 tracking nights per period). This was chosen to ensure tracks are long enough to reflect individual-period space-use and avoid a methodological bias. Notably, although total distance was included as a possible fixed effect in various models within the set, it was not highly ranked and is absent from all leading models.

#### Individual predictability is related to its survival

**Fig. 5:**
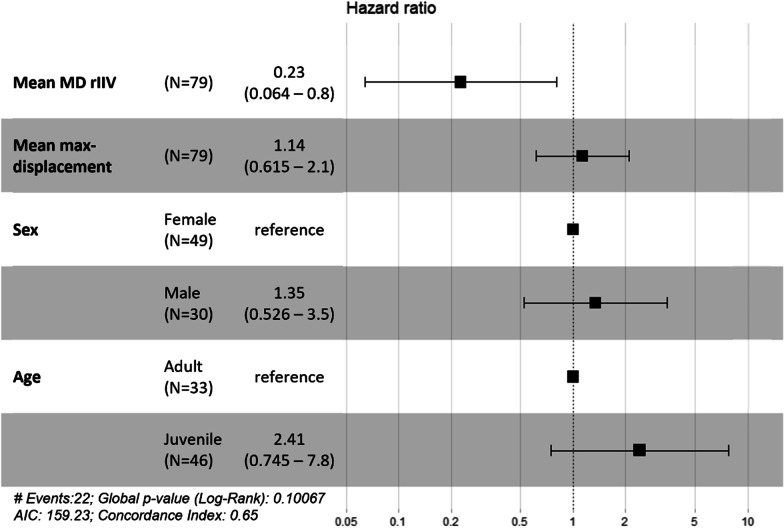
Results of the Cox proportional regression model showing the hazard ratios for tagged barn owls in our system. A hazard ratio < 1 indicates increasing survival probability with the increasing rIIV value (i.e., less predictable individuals surviving longer). A hazard ratio > 1 indicates an increase in hazard and a lower survival probability. For instance, juvenile owls compared to the reference group of adults show a trend in this direction

In agreement with the hypothesis that survival will be related with predictability [H2.2], we find that rIIV of max-displacement is negatively and significantly associated with risk level in the Cox hazard regression. This negative effect means that individuals with higher rIIV values (i.e., less predictable) seem to survive longer, while more predictable individuals (lower rIIV values) are more prone to mortality (Fig. 5; Additional file 1: Table S1). None of the other predictors (sex, age group, max displacement, tracking duration per period) significantly affected regression results and owls’ survival.

While age-dependent diminishing mortality risk is commonly observed in many biological systems—barn owls included [72]—this general pattern is only weakly reflected in our data. Juveniles showed a (non-significant) trend toward higher hazard compared to adults (the reference category) and this modeling result is also reflected in actual mortality rates (10/33 adults vs. 12/46 juveniles died during the study). Presumably, two factors may contribute to this relatively high adult mortality: first, dispersing juveniles readily leave the region (resulting in ‘censored’ status). Indeed, tracking duration for juveniles was marginally shorter than for adults (127.9 ± 92.7 vs. 172 ± 112.3, respectively; *T*_*df* = 60.6_ = 1.84, *p* = 0.06). Second, anthropogenic factors (mostly collisions with cars) that are disproportionately hitting adults (66% of car hits were of adults although they were only 42% of tracked individuals), presumably since they engage in more intensive foraging while providing chicks compared to juveniles that have no dependents.

### Factors affecting individual predictability

**Fig. 6:**
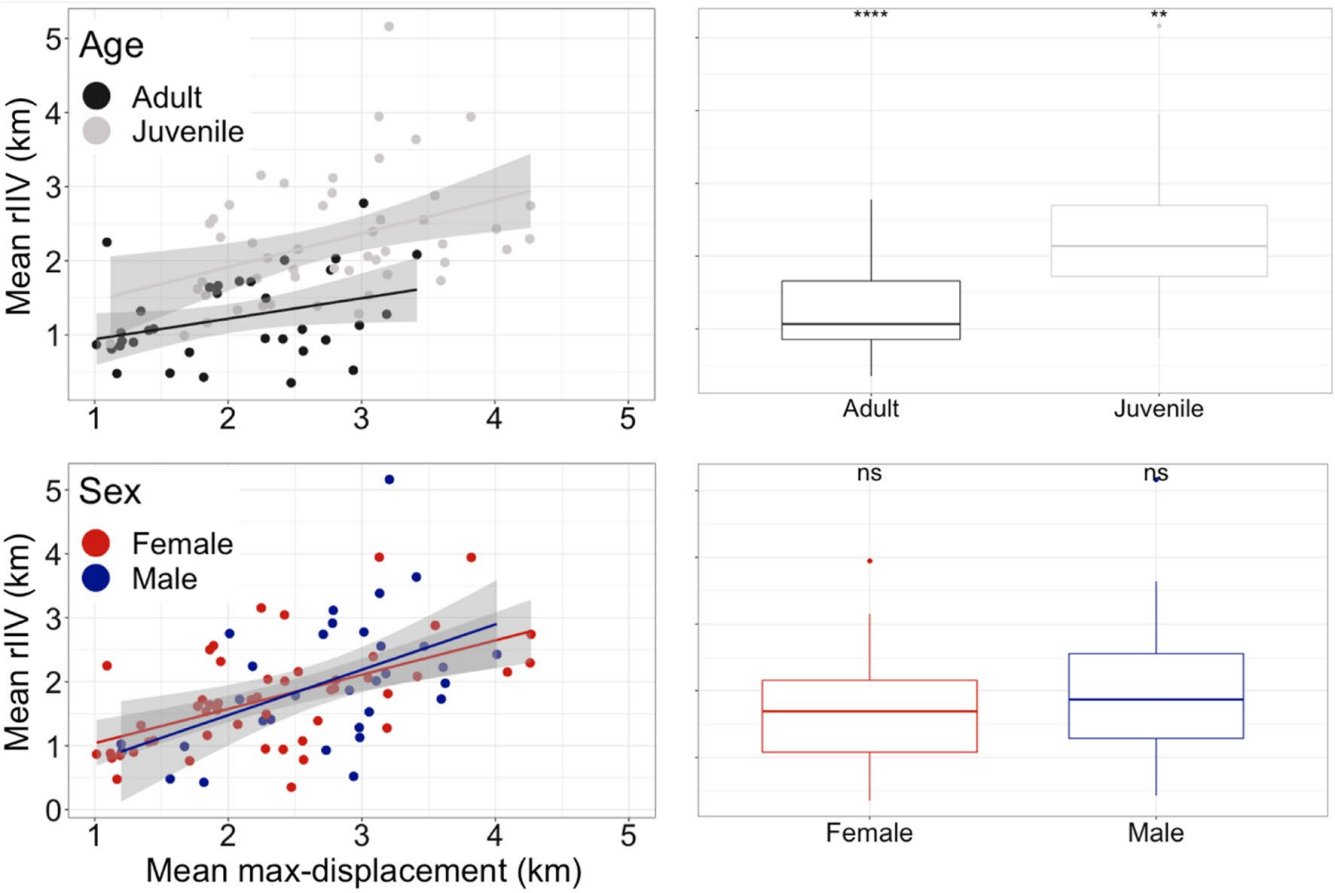
Factors affecting among-individual differences in rIIV. Panels on the left show a positive correlation between the mean and the intra-individual variation (rIIV) of max-displacement split by age (upper left) and sex (lower left). Individuals that move more are less predictable with higher rIIV. Panels on the right show a simple box plot for age (upper right) and sex (lower right). The asterisk reflects results from a Two-way ANOVA indicating that age but not sex affect rIIV

Our last hypothesis [H3.1] that predictability depends on age and sex was only partially supported. The data show that rIIV depends on max-displacement and the age group (Fig. 6), with barn owls becoming predictable with age (aka ‘behavioral canalization’). A direct comparison reveals that juveniles are less predictable than adults with an average value of rIIV of 2.25 ± 0.83 km versus 1.23 ± 0.59 km. In contrast to our expectation, sex did not affect rIIV, and females and males have average rIIV values of 1.74 ± 0.81 km and 1.98 ± 1.02 km, respectively (two-way ANOVA for sex *F*_*df* = 1_ = 1.57, *p* = 0.21; and for age *F*_*df* = *1*_ = 44.05, *p* < 0.001). This basic result is also supported by model comparison, showing that 21 models gained sufficient support (Delta_AICc < 4; Additional file 1: Table S8; out of the 1024 model combination considered). Age was included in all of these models and was the only predictor which significantly affected rIIV (0.856 ± 0.166, *p* < 0.001; Table 2), implying that barn owls become more predictable as they mature. Year and elevation had some support for an effect on rIIV (0.22 ± 0.21 and 0.13 ± 0.14, respectively; Table 2), but weaker and not significant. All other predictors (e.g., latitude, longitude, period, density and sex) had practically no effect (all < 0.1; Additional file 1: Table S9).

**Table 2:** Model-averaged coefficients (full) for fixed effect in the models predicting predictability (rIIV) of barn owls

	Estimate	SE	Adj SE	Z value	Pr ( >|z|)	
(Intercept)	1.047	0.214	0.215	4.868	1.1e−06	***
Age—juvenile	0.856	0.166	0.168	5.101	3.0e−07	***
Elevation	0.134	0.141	0.141	0.949	0.343	
Year-2021	0.221	0.21	0.21	1.050	0.294	

Reference categories for categorical variables were Adult (age group), 2020 (year), Female (sex) and Period 1 (tracking periods)

## Discussion

In this study, we have tested individuality and predictability in barn owl movement. We have tracked barn owls at high resolution over extended periods (fixes every few seconds, tracks of several months) via the cutting-edge ATLAS system. This rich dataset included 74 individuals and revealed clear patterns of individualism in movement. Individuals differed consistently from each other both in their mean movement indices (max-displacement, total distance) and in their residual intra-individual variation (rIIV), a measure of (un)predictability. Some individuals were highly predictable while others were more variable around their mean nightly movement, and this trait was repeatable across periods (4 months-long sessions used in our analyses). Importantly, these two axes of spatial behavioral types (BTs, namely mean movement and predictability level) are not only distinguishing different conspecifics, but they are also associated with apparent ecological consequences: more predictable individuals (low rIIV) have smaller home-ranges (HRs) and lower survival rate. The effects of predictability on HR and survival are above-and-beyond the commonly studied effects of an individual's max-displacement (positively affecting HR size), age (juveniles have larger HR but similar survival in our dataset), sex (no strong effect on HR or survival) and several indices of the local environment (e.g., elevation, coordinates and local density). We also found that age explained some of the differences among individuals’ predictability, with juveniles being largely less predictable than adults, while neither sex nor the local environment strongly affected individuals’ predictability.

To the best of our knowledge, these links are largely under-explored in the literature of movement ecology. Indeed, there is a growing recognition in the importance of spatial-BTs for various ecological properties [10, 17, 23], yet only a few studies have quantified the predictability of individuals in their movement patterns over time in natural settings (e.g., [38]). Further, almost none has shown that predictability can actually relate (or even influence) broader ecological patterns or explored what determines individual predictability in movement. Below we discuss the ecological importance of predictability, compare our findings to similar systems and point out some possible future directions.

### Spatial behavioral types (BTs) and movement predictability

Consistent among-individual behavioral variation (aka personality or BTs) has become mainstream in behavioral ecology [73]. More recently, approaches developed in behavioral ecology have been adopted to study individuality in movement and spatial-BTs [10, 12]. Indeed, movement and space use behaviors show relatively high repeatability (~ 0.67; [17]) compared to the mean level of other behaviors (Rp = 0.37; [73]). These higher values reflect the fact that individuals are repeatedly “assayed” in different environments. The observed differences among them reflect, at least to some extent, the environmental heterogeneity among their HRs [74, 75]. Our repeatability estimates for the barn owl movements fall on the lower side of this range (R < 0.4). This lower value might reflect a considerable HR overlap among many of our individuals, tracked within a relatively small area. Thus, owls might be experiencing relatively similar conditions that reduce the contribution of spatial heterogeneity to apparent repeatability of their movement. The poor predictive power of the ecological predictors we considered for HR and movement (distance from the system's center, the coordinates, elevation and owls’ density) supports this explanation.

Studies investigating predictability in movement are particularly rare, even though movement data is very suitable for such investigation by offering numerous repeated “assays” (e.g., days) for each tracked individual [38, 39]. Here we have used merely two indices of movement (max displacement and total distances), but numerous other indices can be relevant. For instance, Michelangeli et al. [23] have used several indices (including these two) in order to demonstrate that sleepy lizards (*Tiliqua rugosa*) have distinct spatial-BTs, and that some indices co-vary (i.e., movement syndromes). Because some of these commonly used movement indices (e.g., total tracking distance) are sensitive to sampling rate [76], it is essential to unify rates across individuals (or to subsample some for unifying it). Future studies may sub-sample larger datasets like the one included here to directly test the sensitivity of predictability estimates of different movement indices, or of their combined scores (e.g., via principal component analysis), to the properties of tracking datasets (e.g., resolution, duration, number of individuals).

Broadly speaking, quantifying predictability from movement tracks should be more accurate when using high resolution data, simply because it improves the estimation of the track properties. Our data is of exceptionally high temporal resolution (a fix every ~ 8 s), but we suggest that slightly lower resolutions (e.g., fixes every few minutes) should still be suitable for quantifying predictability as they are quite reliably daily movement patterns. Further, predictability estimates for other temporal scales (e.g., weekly, seasonally, yearly) might be highly relevant for other systems, depending on the duration and resolution of the tracking data at hand. Even coarse resolution data might still be suitable for space-use patterns (e.g., seasonal HR; [18]) but probably less for precise descriptions of the daily movements as done here or for brown bears (*Ursus arctos*) [39]. Given that high-resolution datasets with tracking of numerous individuals over longer periods are becoming more readily available thanks to improving technologies [25, 48], we suggest that quantifying spatial-BTs and specifically movement predictability will be very feasible for many more study systems. These detailed tracking datasets, along with complementary sensor data (e.g., accelerometers) will allow more scholars to address individuality of movement, and investigate the factors that shape it, and its consequences for individuals and species across space and time [24].

### The ecological importance of movement predictability

#### An association with home-range size

The ecological or evolutionary consequences of BTs are well acknowledged for various systems and contexts [77, 78]. Less is known about the implications of spatial-BTs, and the association of their fine-scale movements (e.g., daily indices) with broader space use patterns at the HR scale. Accumulating examples demonstrate that variation in HR size is repeatable, and that it can be linked to independently measured BTs (e.g., [18, 79, 80]), or to spatial-BTs assess from the movement data itself [19, 23]. Arguably, local and global scales are expected to co-vary in indices like the ones used here (namely max-displacement), with individuals that move more at the daily scale also covering larger HRs. Indeed, we also find this positive effect of max displacement on HR size (Fig. 4A).

We did not find an effect of sex on HR size. Interestingly, previous studies of barn owls' HR reported contradicting results. Séchaud et al. [81] found smaller male HR sizes during the breeding season (but tracked individuals for up to two weeks only), while Roulin [41] report a larger HR for males for the same season. Our null result might reflect either a true difference in the biology between the systems; or the longer scope of our tracking (> 25 days per period) where seasonal changes across the annual cycle mask the differences during breeding season; or due to differences in home-range estimators used for calculation [82]. Finally, geographical and other demographic factors had hardly any effect on HR size.

Importantly, even while accounting for other confounding factors (including max displacement), we discover that predictability per se was a strong predictor of HR size, with an effect size of almost a half of the max-displacement (Fig. 4B). We find that unpredictable individuals (high rIIV) have larger HRs (even within a cohort and sex group). This finding might reflect more consistent and repeatable use of the same locations and is in agreement with previously described syndromes in movement patterns [17, 22, 23]. Future studies may explore if predictable individuals (in our single index of max displacement) tended to also do their longer movement in the same locations, and directions further contributing to smaller HRs of these individuals. Unpredictable individuals, in contrast, might also show lower predictability in where, when and in what direction they do their long movement, revealing further structures in barn owl movements.

More generally, if the strong association between individual predictability and its HR is generalizable beyond our focal population (regardless of the specific mechanism proposed here), this could provide an insightful currency that does not require additional data collection, but merely to include this aspect in relevant models of the existing tracking data. Given the high relevance of accurate HR estimates for diverse questions in ecology and conservations [58, 83], adding this predictive power can facilitate ecological application and improve the prediction accuracy of future models. For instance, HR overlap among neighbors is often important for disease and parasite transmission [84, 85]. Future studies can also further explore possible effects of population density (that had no effect in this system) and HR overlap on behavioral movement indices of individuals and their predictability.

#### Consequences for survival

Studying behavioral predictability in the wild might reveal life history trade-offs which are not present in laboratory studies [86]. Only a handful of studies quantified IIV in situ permitting investigation of the ecological significance of predictability. Examples include IIV in aggressiveness of male deer affecting their mating success [35]; predictability affecting foraging site fidelity of kittiwakes [87], and brown bear movement predictability potentially affecting their ability to cope with changes in the environment [39]. Such studies demonstrate various potential ecological implications of IIV, but to the best of our knowledge, none of the previous examples have demonstrated that predictability affected survival, which is arguably the cornerstone of individual fitness.

Our long tracking and large sample size allowed us to identify the fate of many individuals, with considerable mortality rates during the study. We find that predictable individuals survive less. This might appear counterintuitive since predictability can be preserved as a risk-averse behavior. Yet, this interpretation for our context (simply higher rIIV in max displacement) is not accurate. Unpredictable movement can in fact reflect the adaptive response to changing environmental conditions that were unmeasured (hence adaptive plasticity). It can also reflect exploration bouts with long flights (as evident also in Additional file 1: Fig. S2) providing information and access to spatially different resources [88]. Further, although individuals expressing more “risky” behaviors should in-theory suffer from higher mortality, bolder BTs were sometimes found to live significantly longer in the wild (in contrast to the laboratory), suggesting that apparently risky behavior in one context can be beneficial in others [86]. Such findings agree with our result that unpredictable barn owls have a higher survival rate.

### Factors affecting predictability

Identifying what determines individual’s BT, and here specifically its predictability in movement is essential for a mechanistic understating of this phenomenon and its ecological consequences as well as for generalizing across systems. Variation in predictability can reflect both proximate and ultimate reasons. At the proximate level, we find that older individuals are more predictable than young ones. This was demonstrated before for the predictability of other behaviors [33, 38, 43]. Common explanations for such ‘canalization’ include the idea that with increased exposure to predictable cues, individuals may become more certain in their assessment of the environment allowing traits to become fixated. Other suggested explanations have to do with traits being linked to physiological factors such as growth rate and hormonal profiles, and behaviors change concurrently with these physiological reorganizations.

Observed consistent behavioral differences among individuals recorded under natural conditions may not reflect only inherent differences in spatial-BTs, but also arise from (at least partially) differences in the geographical features or resource distribution [17, 75]. Here we do not find support for this general pattern in explaining variation in predictability. In our study system, there is an elevation gradient (from + 100 m ASL at the west, down to − 250 m BSL in the east), affecting local temperature, soil type and crop type, which in turn determine food availability (abundance and species composition of the rodent community). Yet, variation in predictability was not associated with the gradient (Additional file 1: Fig. S2) or other geographical factors, and more/less predictable individuals were apparently randomly distributed along the region, supporting the argument that our measured behavior results (mostly) from a genuine inherent behavioral tendencies and preferences of individuals. In the future, experimental approaches (e.g., relocation) can reveal the distinct contributions of background heterogeneity and the individual tendency to spatial-BTs [75].

On a more ultimate level, differences in spatial-BTs can arise from several reasons including individual diet specialization—variations in preferences for certain habitat features, resources (a particular type of food), and/or social interactions [89]. While some of these are likely consistent at the individual level [90] others will depend on the environmental dynamics. In the future, studies can explore if differences in predictability are associated with diet preferences, and what mechanisms generate apparent differences in predictability that remain repeatable over time. One may ask whether the shape of the distribution of the index in mind (here max displacement) changes. Namely, this asks if unpredictable individuals just have larger variation, or do they have a fat-tailed distribution. Indeed, here we find support for the latter, with high rIIV values appearing through very long-range displacement in a small subset of nights (Additional file 1: Fig. S3), and not through a homogenous larger variation. Whether these long-range movements reflect a distinct behavioral mode expressed only in certain individuals (and why) remains unknown. Such modes can be inferred from modeling step size and turning angle distributions, allowing identification of different subgroups in the population [91, 92]. Applying such an approach to spatial-BTs of different predictabilities may offer a strong integration of existing tools from movement ecology with new concepts from behavioral ecology.

### Concluding remarks

In this initial study we only explored spatial-BTs in three traits (travel distance, max-displacement, and predictability of the latter). Yet, future studies of spatial-BTs and spatial syndromes can expand to various other movement indices. These may include classical indices of movement (e.g., tortuosity; [22, 23]) and space use [17, 93], as well as newer indices such as movement openness and diameter [36]. Similarly, other factors affecting rIIV beyond those explored here (sex, age, year, season and a few local environmental indicators) can be investigated. As well as other ecological and evolutionary consequences (beyond HR size and survival addressed here) can be studied. Future topics may include the implications of spatial-BT for parasite prevalence [94], or breeding success [35].

Finally, our tracking is biased toward owls staying within the ATLAS region. In our study, 20 out of the 45 juveniles left the study area (for at least 24 h), yet most of them returned after some absence. If individuals with remarkably different spatial-BTs choose to leave the region completely [95] then our sample might be underestimating the variation in the population. Despite these limitations, we were able to describe the prevalence of spatial-BTs and consistent variation in individual predictability within our population. We also validated the ecological relevance of predictability to their fitness (via survival) and broader space use (HR size). Unpredictable individuals may cope better under changing environmental conditions [39]. Human-induced environmental changes act as a non-random filter selecting species capable of coping with such changes. Likewise, environmental changes can also affect intraspecific composition, with predictability in movement indices as key axes that is still poorly explored [28, 32]. Understanding the scope and role of intraspecific variation in movement and other spatial behaviors will help us better conserve species, their habitats, and their ecological roles (e.g., [10, 96]) in the face of unprecedented environmental change.

## Supplementary Information

**Additional file 1: Table S1**. Results of the Cox proportional regression model without random factor. **Table S2**. Results of the Cox proportional regression model with ring ID as a random factor. **Table S3**. Number of individuals tracked each period. **Table S4**. Tracking periods, ring ID, sex and age for all individuals in the dataset. **Table S5**. Repeatability (Rp) and coefficient of variation for among-individual variance (CVi) values for total-distance and nightly max-displacement. **Table S6**. Modified Akaike Information Criterion (AICc) for models estimating HR size by all our fixed effects. **Table S7**. Fixed effect estimation on the home-range (HR) size from the best-ranked model. Reference categories for categorical variables were Adult (age group) and Period 1 (tracking periods). **Table S8**. Models estimating predictability (rIIV) by all our fixed effects. **Table S9**. Model-averaged coefficients (full) for fixed effect in the models predicting predictability (rIIV) of barn owls. **Figure S1**. Age and sex-related differences in the nightly max-displacement for the entire dataset (not divided by periods). Juveniles have longer distances compared to adults, and males tend to have longer distances compared to females (one-way ANOVA, F_*df* = 3_ = 8.97, *P* < 0.001). **Figure S2**. Barn owls' central home-range (HR) and rIIV spatial scattering across the study area of the Harod valley, for adults (**A**) and juveniles (**B**). Each circle represents a single individual, where the center and the radius of the circle represent the HR center and size (log HR size, by addCircleMarkers function), respectively. Colors indicate each individual's predictability value (i.e., mean rIIV, splits into five groups). **Figure S3**. Histogram of nightly max-displacement values for the five most unpredictable individuals (top, red) and five most predictable individuals (bottom, blue) for the second period of 2021. Both groups were generally characterized by a well-defined peak around 3–5 km, but unpredictable individuals had occasionally long values of max-displacement contributing to their higher rIIV values

## Data Availability

The raw data will be published in Movebank.

## Abbreviations

AICc: Corrected Akaike information criterion
BT: Behavioral types
CVi: Coefficient of variation for among-individual variance
DHGLM: Double-hierarchical generalized linear model
GPS: Global positioning system
HR: Home-range
ID: Identification
(r)IIV: (Residual) intra-individual variation
Rp: Repeatability
STD: Standard deviation
