# Supplementary material for: Movement predictability of individual barn owls facilitates estimation of home range size and survival
Source: Mov Ecol. 2023 Feb 7;11:10. doi: 10.1186/s40462-022-00366-x (PMC9906850; doi:10.1186/s40462-022-00366-x)
Supplement: Supplementary file 1 — Additional file 1: Table S1. Results of the Cox proportional regression model without random factor. Table S2. Results of the Cox proportional regression model with ring ID as a random factor. Table S3. Number of individuals tracked each period. Table S4. Tracking periods, ring ID, sex and age for all individuals in the dataset. Table S5. Repeatability (Rp) and coefficient of variation for among-individual variance (CVi) values for total-distance and nightly max-displacement. Table S6. Modified Akaike Information Criterion (AICc) for models estimating HR size by all our fixed effects. Table S7. Fixed effect estimation on the home-range (HR) size from the best-ranked model. Reference categories for categorical variables were Adult (age group) and Period 1 (tracking periods). Table S8. Models estimating predictability (rIIV) by all our fixed effects. Table S9. Model-averaged coefficients (full) for fixed effect in the models predicting predictability (rIIV) of barn owls. Figure S1. Age and sex-related differences in the nightly max-displacement for the entire dataset (not divided by periods). Juveniles have longer distances compared to adults, and males tend to have longer distances compared to females (one-way ANOVA, Fdf = 3 = 8.97, P < 0.001). Figure S2. Barn owls' central home-range (HR) and rIIV spatial scattering across the study area of the Harod valley, for adults (A) and juveniles (B). Each circle represents a single individual, where the center and the radius of the circle represent the HR center and size (log HR size, by addCircleMarkers function), respectively. Colors indicate each individual's predictability value (i.e., mean rIIV, splits into five groups). Figure S3. Histogram of nightly max-displacement values for the five most unpredictable individuals (top, red) and five most predictable individuals (bottom, blue) for the second period of 2021. Both groups were generally characterized by a well-defined peak around 3–5 km, but unpredictable individuals [file 40462_2022_366_MOESM1_ESM.docx]

## Appendix- additional results

### Factors affecting predictability

To further validate the results presented in the main text of this section we repeated the analysis with a second DHGLM. In which, all effects (and not only the individual identity) were included in the model of the residuals in addition to the mean model. We modeled nightly max-displacement as a response variable with sex and age group as fixed effects and individual identity as a random effect. This model shows that individuals differ in how predictable they are in their nightly movement and that juveniles are less predictable in their behavior than adults, while sex has no significant effect on predictability.

### Tabled and figures

**Table S1.** Results of the Cox proportional regression model without random factor. Reference categories for categorical variables were Female (sex) and Adult (age group). Likelihood ratio test=7.76 on 4 df, p=0.1007.

| Fixed effect included in the model | coef | exp(coef) | se(coef) | z | p |
| --- | --- | --- | --- | --- | --- |
| Sex - male | 0.299 | 1.348 | 0.481 | 0.622 | 0.534 |
| Age - juvenile | 0.878 | 2.406 | 0.597 | 1.469 | 0.142 |
| Mean max-displacement | 0.127 | 1.136 | 0.313 | 0.408 | 0.683 |
| Mean max-displacement rIIV | -1.482 | 0.227 | 0.645 | -2.299 | 0.021 |

**Table S2.** Results of the Cox proportional regression model with ring ID as a random factor. Reference categories for categorical variables were Female (sex) and Adult (age group). Integrated loglik: Chisq=7.79; df=5; p=0.15. Random effect Ring_ID Intercept: Std Dev=0.553; Variance=0.3.

| Fixed effect included in the model | coef | exp(coef) | se(coef) | z | p |
| --- | --- | --- | --- | --- | --- |
| Sex - male | 0.369 | 1.447 | 0.527 | 0.70 | 0.48 |
| Age - juvenile | 0.917 | 2.502 | 0.632 | 1.45 | 0.15 |
| Mean max-displacement | 0.080 | 1.083 | 0.343 | 0.23 | 0.82 |
| Mean max-displacement rIIV | -1.519 | 0.219 | 0.660 | -2.23 | 0.021 |

**Table S3.** Number of individuals tracked each period. Periods are 1st-period Feb-May, incubating/nesting period; 2nd-period Jun-Sep, rearing/post-breeding period; and 3rd-period Oct-Jan, fall-winter time.

| Period | Adult female | Adult male | Juvenile female | Juvenile male | Total |
| --- | --- | --- | --- | --- | --- |
| 2020 period 2 | 5 | 1 | 11 | 9 | 26 |
| 2020 period 3 | 2 | 2 | 5 | 8 | 17 |
| 2021 period 1 | 10 | 5 | 3 | 2 | 20 |
| 2021 period 2 | 20 | 6 | 12 | 10 | 48 |
| 2021 period 3 | 12 | 2 | 1 | 2 | 17 |
| Total | 49 | 16 | 32 | 31 | 128 |

**Table S4.** Tracking periods, ring ID, sex and age for all individuals in the dataset. Periods are 1st-period Feb-May, incubating/nesting period; 2nd-period Jun-Sep, rearing/post-breeding period; and 3rd-period Oct-Jan, fall-winter time.

| Ring | sex | age | 2020_peri2 | 2020_peri3 | 2021_peri2 | 2021_peri3 | 2021_peri1 |
| --- | --- | --- | --- | --- | --- | --- | --- |
| G16385 | male | Juvenile | V | V |  |  |  |
| G16386 | male | Juvenile | V |  |  |  |  |
| G16387 | male | Juvenile | V | V |  |  |  |
| G16388 | male | Juvenile | V | V |  |  |  |
| G18092 | male | Juvenile |  |  | V |  |  |
| GG26748 | female | adult |  |  | V | V |  |
| GG29111 | female | adult |  |  | V | V |  |
| GG30813 | male | adult |  |  | V |  | V |
| GG34536 | female | adult | V |  |  |  |  |
| GG34680 | female | adult | V |  |  |  |  |
| GG35107 | female | adult |  |  |  |  | V |
| GG36056 | female | adult |  |  | V | V |  |
| GG37001 | male | Juvenile | V | V |  |  |  |
| GG37003 | male | Juvenile | V |  |  |  |  |
| GG37004 | female | Juvenile | V |  |  |  |  |
| GG37005 | female | Juvenile | V |  |  |  |  |
| GG37017 | female | Juvenile | V |  |  |  |  |
| GG37018 | female | Juvenile | V |  |  |  |  |
| GG37019 | female | Juvenile | V | V |  |  | V |
| GG37019 | female | adult |  |  | V |  |  |
| GG37020 | male | Juvenile | V |  |  |  |  |
| GG37048 | female | adult | V |  |  |  |  |
| GG37049 | female | Juvenile | V | V |  |  |  |
| GG37055 | male | Juvenile | V | V |  |  | V |
| GG37088 | male | adult |  |  |  |  | V |
| GG37106 | female | adult |  |  | V | V | V |
| GG37113 | female | adult | V | V | V | V | V |
| GG37114 | male | adult | V |  |  |  |  |
| GG37121 | female | adult | V | V |  |  |  |
| GG37128 | male | Juvenile |  | V |  |  | V |
| GG37128 | male | adult |  |  | V | V |  |
| GG37130 | male | Juvenile |  | V |  |  |  |
| GG37131 | female | Juvenile |  | V |  |  | V |
| GG37131 | female | adult |  |  | V | V |  |
| GG37133 | male | adult |  | V |  |  | V |
| GG37136 | female | adult |  |  | V |  | V |
| GG37138 | female | adult |  |  | V |  | V |
| GG37139 | male | adult |  |  | V |  | V |
| GG37140 | female | adult |  |  | V | V | V |
| GG37141 | female | adult |  |  | V | V | V |
| GG37156 | female | Juvenile |  |  | V |  |  |
| GG37157 | female | Juvenile |  |  | V |  |  |
| GG37163 | female | adult |  |  | V |  | V |
| GG37172 | female | adult |  |  | V |  |  |
| GG39117 | female | adult |  |  | V |  | V |
| GG39226 | male | adult |  |  | V |  |  |
| GG39262 | female | adult |  |  | V | V | V |
| GG39394 | female | Juvenile | V | V |  |  |  |
| GG39394 | female | adult |  |  | V |  |  |
| GG39395 | female | Juvenile | V | V |  |  | V |
| GG39395 | female | adult |  |  | V | V |  |
| GG39396 | female | Juvenile | V |  |  |  |  |
| GG39398 | female | Juvenile | V |  |  |  |  |
| GG39405 | female | Juvenile | V |  |  |  |  |
| GG39406 | male | Juvenile | V | V |  |  |  |
| GG41258 | female | adult |  |  | V | V |  |
| GG41259 | female | adult |  |  | V |  |  |
| GG41260 | female | Juvenile |  |  | V |  |  |
| GG41269 | male | Juvenile |  |  | V |  |  |
| GG41283 | male | Juvenile |  |  | V |  |  |
| GG41284 | female | Juvenile |  |  | V |  |  |
| GG41285 | female | Juvenile |  |  | V |  |  |
| GG41287 | female | Juvenile |  |  | V |  |  |
| GG41291 | female | Juvenile |  |  | V |  |  |
| GG41301 | male | Juvenile |  |  | V |  |  |
| GG41303 | male | Juvenile |  |  | V |  |  |
| GG41305 | female | Juvenile |  |  | V |  |  |
| GG41309 | male | Juvenile |  |  | V |  |  |
| GG41310 | male | Juvenile |  |  | V | V |  |
| GG41312 | male | Juvenile |  |  | V | V |  |
| GG41317 | male | Juvenile |  |  | V |  |  |
| GG41318 | female | Juvenile |  |  | V |  |  |
| GG41336 | female | Juvenile |  |  | V |  |  |
| GG41339 | female | Juvenile |  |  | V | V |  |
| GG41340 | female | adult |  |  | V | V |  |
| GG41341 | male | Juvenile |  |  | V |  |  |
| GG41344 | female | Juvenile |  |  | V |  |  |
| GG53006 | male | adult |  |  | V | V |  |

**Table S5.** Repeatability (Rp) and coefficient of variation for among-individual variance (CVi) values for total-distance and nightly max-displacement.

|  | Rp | CVi |
| --- | --- | --- |
| Total distance | 0.391 [0.302, 0.483] | 0.526 [0.441, 0.611] |
| Max displacement | 0.228 [0.158, 0.297] | 0.362 [0.303, 0.426] |

**Table S6.** Modified Akaike Information Criterion (AICc) for models estimating HR size by all our fixed effects. All models include (i) **Age**; (ii) **Period**; and **TrackingDuration** (the number of tracking nights for each period, to account for tracking duration) as fixed effects, as well as additional fixed effects which are mentioned in detail. Abbreviations: **MaxDisp**- mean nightly maximal displacement; **rIIV**- the index of predictability in max-displacement, mean value; **DensScore-** the number of occupied boxes within a radius of 1.7 km (the median HR’s radius) from the HR center along the study years; **Dist**- distance of HR center from the system's center; and HR center: **Lon**- longitude coordinates; **Lat**- latitude coordinates; **Elev**- elevation (m).

| Fixed effect included in the model | K | AICc | Delta_AICc | AICcWt | Cum.Wt | LL |
| --- | --- | --- | --- | --- | --- | --- |
| MaxDisp; rIIV | 9 | 311.04 | 0 | 0.23 | 0.23 | -145.74 |
| MaxDisp; rIIV; Dist | 10 | 311.94 | 0.9 | 0.15 | 0.37 | -145 |
| MaxDisp; rIIV; Lat | 10 | 312.28 | 1.24 | 0.12 | 0.5 | -145.18 |
| MaxDisp; rIIV; Elev | 10 | 312.61 | 1.57 | 0.1 | 0.6 | -145.34 |
| MaxDisp; rIIV; DensScore | 10 | 312.82 | 1.78 | 0.09 | 0.69 | -145.44 |
| Sex; MaxDisp; rIIV | 10 | 313.37 | 2.33 | 0.07 | 0.76 | -145.72 |
| MaxDisp; rIIV; Lon | 10 | 313.4 | 2.36 | 0.07 | 0.83 | -145.73 |
| Sex; MaxDisp; rIIV; Dist | 11 | 314.34 | 3.3 | 0.04 | 0.88 | -145 |
| Sex; MaxDisp; rIIV; Lat | 11 | 314.6 | 3.56 | 0.04 | 0.92 | -145.13 |
| Sex; MaxDisp; rIIV; Elev | 11 | 314.93 | 3.89 | 0.03 | 0.95 | -145.29 |
| Sex; MaxDisp; rIIV; DensScore | 11 | 315.19 | 4.15 | 0.03 | 0.98 | -145.43 |
| Sex; MaxDisp; rIIV; Lon | 11 | 315.77 | 4.73 | 0.02 | 1 | -145.71 |
| MaxDisp | 8 | 322.52 | 11.48 | 0 | 1 | -152.64 |
| MaxDisp; Dist | 9 | 323.7 | 12.66 | 0 | 1 | -152.07 |
| MaxDisp; DensScore | 9 | 324.11 | 13.07 | 0 | 1 | -152.27 |
| MaxDisp; Lat | 9 | 324.63 | 13.59 | 0 | 1 | -152.53 |
| MaxDisp; Lon | 9 | 324.8 | 13.76 | 0 | 1 | -152.62 |
| MaxDisp; Elev | 9 | 324.85 | 13.81 | 0 | 1 | -152.64 |

**Table S7.** Fixed effect estimation on the home-range (HR) size from the best-ranked model. Reference categories for categorical variables were Adult (age group) and Period 1 (tracking periods).

|  | Estimate | Std. Error | t. value |
| --- | --- | --- | --- |
| (Intercept) | 15.69 | 0.26 | 58.90 |
| Age - juvenile | 0.35 | 0.20 | 1.75 |
| Period - peri2 | 0.51 | 0.22 | 2.26 |
| Period - peri3 | 0.43 | 0.25 | 1.75 |
| Tracking duration | -0.003 | 0.002 | -1.39 |
| Mean max-displacement | 0.49 | 0.09 | 5.18 |
| Mean MaxDisp rIIV | 0.27 | 0.10 | 2.66 |

**Table S8.** Models estimating predictability (rIIV) by all our fixed effects. Ranking based on ΔAICc of top models (<4) comparing fixed effect combination affecting rIIV. Abbreviations: **TrackingDuration**- the number of tracking nights for each period, to account for tracking duration); and HR center: **Lon**- longitude coordinates; **Lat**- latitude coordinates; and **Elevation** (m).

| Fixed effect included in the model | df | logLik | AICc | Delta_AICc | AICcWt |
| --- | --- | --- | --- | --- | --- |
| Age–elevation-year | 6 | -140.08 | 292.88 | 0.00 | 0.09 |
| Age–elevation-tracking duration-year | 7 | -139.04 | 293.04 | 0.16 | 0.09 |
| Age–elevation-tracking duration | 6 | -140.74 | 294.19 | 1.31 | 0.05 |
| Age- year | 5 | -141.89 | 294.28 | 1.40 | 0.05 |
| Age–elevation | 5 | -142.01 | 294.53 | 1.66 | 0.04 |
| Age | 4 | -143.12 | 294.58 | 1.70 | 0.04 |
| Age-Lat-year | 6 | -141.06 | 294.83 | 1.95 | 0.04 |
| Age–elevation-tracking duration-Lon-year | 8 | -138.81 | 294.87 | 1.99 | 0.03 |
| Age-Lon-Lat-year | 7 | -139.99 | 294.94 | 2.06 | 0.03 |
| Age-Lat | 5 | -142.62 | 295.75 | 2.88 | 0.03 |
| Age–tracking duration-Lon-year | 7 | -140.40 | 295.76 | 2.88 | 0.03 |
| Age–tracking duration | 5 | -142.67 | 295.83 | 2.96 | 0.03 |
| Age–tracking duration-Lon-Lat-year | 8 | -139.36 | 295.97 | 3.09 | 0.03 |
| Age–tracking duration-year | 6 | -141.67 | 296.05 | 3.17 | 0.03 |
| Age–tracking duration-Lat-year | 7 | -140.70 | 296.36 | 3.48 | 0.03 |
| Age-Lon-Lat | 6 | -141.84 | 296.40 | 3.52 | 0.03 |
| Age–elevation–tracking duration-Lon | 7 | -140.78 | 296.52 | 3.65 | 0.03 |
| Age–elevation–tracking duration -Lat-year | 8 | -139.69 | 296.62 | 3.74 | 0.01 |
| Age–tracking duration-Lat | 6 | -142.03 | 296.76 | 3.89 | 0.01 |
| Age–elevation-sex-year | 7 | -140.93 | 296.81 | 3.93 | 0.01 |
| Age–elevation-Lat-year | 7 | -140.94 | 296.84 | 3.96 | 0.01 |

***Table S9.*** Model-averaged coefficients (full) for fixed effect in the models predicting predictability (rIIV) of barn owls. Reference categories for categorical variables were Adult (age group), 2020 (year), Female (sex) and Period 1 (tracking periods). Abbreviations: **Tracking Duration**- the number of tracking nights for each period; **Density**- the number of occupied boxes within median HR size; and HR center: **Lon**- longitude coordinates; **Lat**- latitude coordinates; and **Elevation** (m).

|  | Estimate | Std.Error | Adj SE | Z value | Pr(>\|z\|) |  |
| --- | --- | --- | --- | --- | --- | --- |
| (Intercept) | 1.047 | 0.214 | 0.215 | 4.868 | 1.1e-06 | *** |
| Age- juvenile | 0.856 | 0.166 | 0.168 | 5.101 | 3.0e-07 | *** |
| Elevation | 0.134 | 0.141 | 0.141 | 0.949 | 0.343 |  |
| Year-2021 | 0.221 | 0.21 | 0.21 | 1.050 | 0.294 |  |
| Tracking duration | -0.072 | 0.091 | 0.091 | 0.790 | 0.429 |  |
| Lat | 0.066 | 0.153 | 0.153 | 0.429 | 0.668 |  |
| Lon | 0.053 | 0.112 | 0.113 | 0.469 | 0.639 |  |
| Sex-male | -0.008 | 0.06 | 0.06 | 0.135 | 0.893 |  |
| Density | 0.003 | 0.03 | 0.03 | 0.096 | 0.923 |  |
| Distance from center | 0.004 | 0.026 | 0.026 | 0.155 | 0.877 |  |
| Period-peri2 | 0.011 | 0.066 | 0.067 | 0.165 | 0.869 |  |
| Period-peri3 | 0.006 | 0.054 | 0.055 | 0.108 | 0.914 |  |


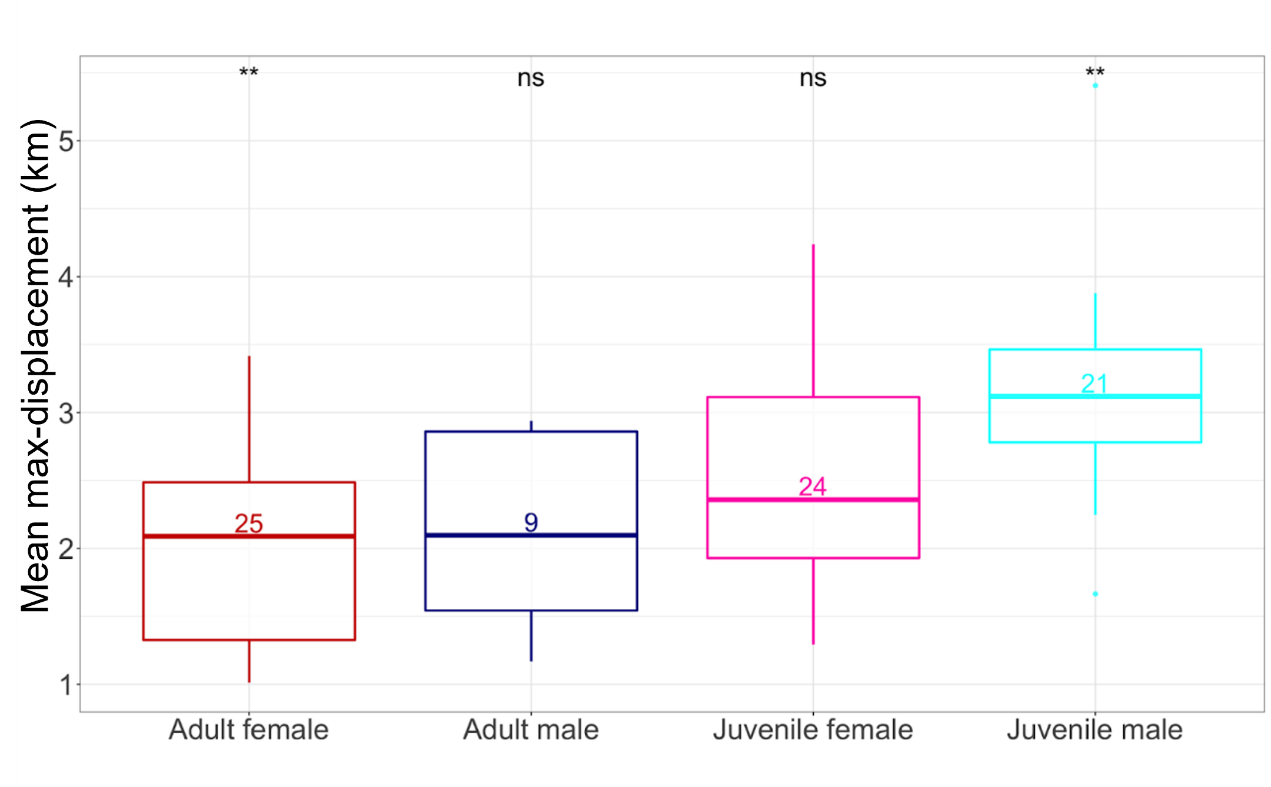


***Figure S1.*** Age and sex-related differences in the nightly max-displacement for the entire dataset (not divided by periods). Juveniles have longer distances compared to adults, and males tend to have longer distances compared to females (one-way ANOVA, F_df=3_=8.97, *P*<0.001).

**
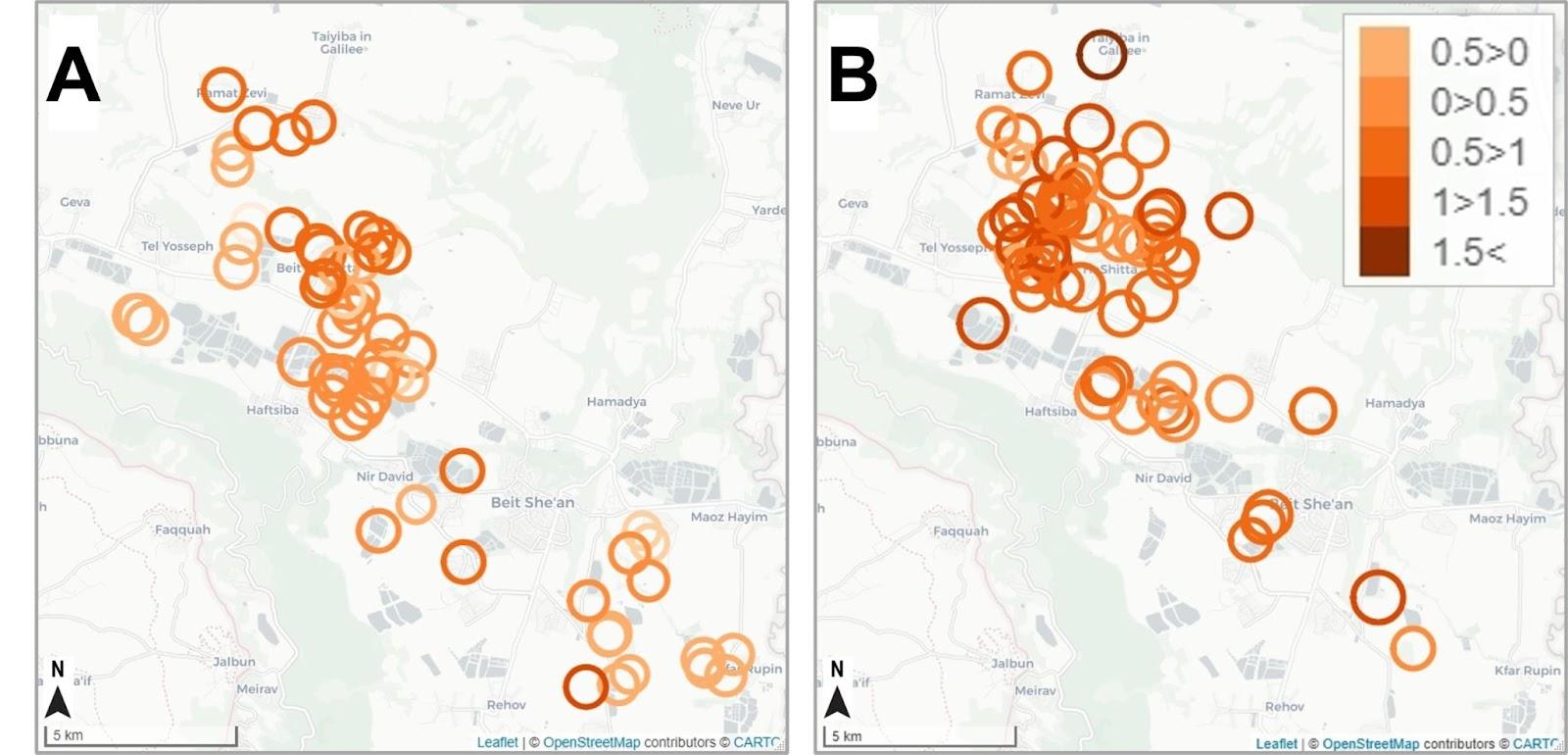
**

***Figure S2.*** Barn owls' central home-range (HR) and rIIV spatial scattering across the study area of the Harod valley, for adults (**A**) and juveniles (**B**). Each circle represents a single individual, where the center and the radius of the circle represent the HR center and size (log HR size, by addCircleMarkers function), respectively. Colors indicate each individual's predictability value (i.e., mean rIIV, splits into five groups).


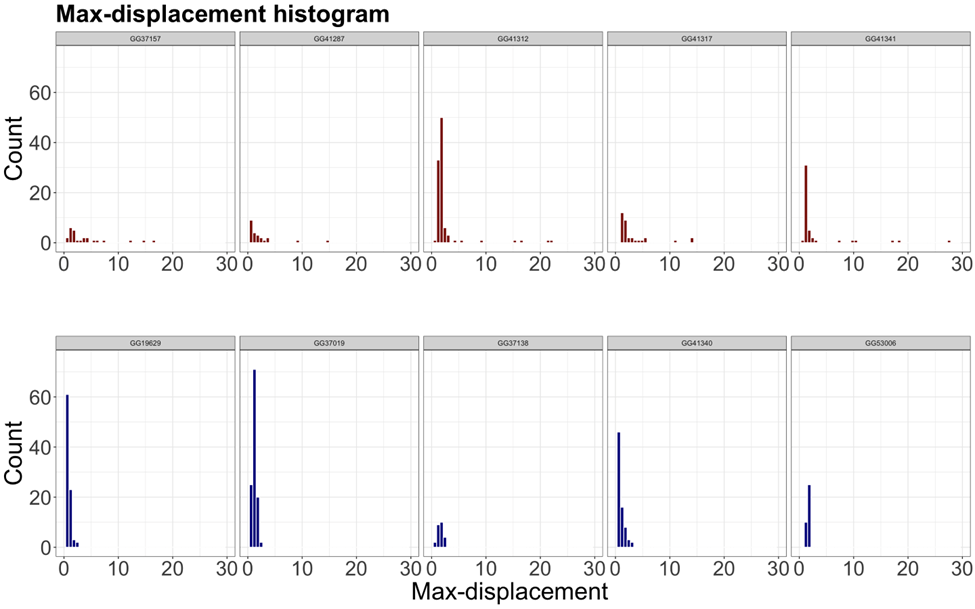


***Figure S3.*** Histogram of nightly max-displacement values for the five most unpredictable individuals (top, red) and five most predictable individuals (bottom, blue) for the second period of 2021. Both groups were generally characterized by a well-defined peak around 3-5 km, but unpredictable individuals had occasionally long values of max-displacement contributing to their higher rIIV values.
